# Essential Emergency and Critical Care: a consensus among global clinical experts

**DOI:** 10.1136/bmjgh-2021-006585

**Published:** 2021-09-21

**Authors:** Carl Otto Schell, Karima Khalid, Alexandra Wharton-Smith, Jacquie Oliwa, Hendry R Sawe, Nobhojit Roy, Alex Sanga, John C Marshall, Jamie Rylance, Claudia Hanson, Raphael K. Kayambankadzanja, Lee A Wallis, Maria Jirwe, Tim Baker, The EECC Collaborators*, Adam Asghar

**Affiliations:** 1 Department of Global Public Health, Karolinska Institutet, Stockholm, Sweden; 2 Centre for Clinical Research Sörmland, Uppsala University, Eskilstuna, Sweden; 3 Department of Internal Medicine, Nyköping Hospital, Nyköping, Sweden; 4 Muhimbili University of Health and Allied Sciences, Dar es Salaam, United Republic of Tanzania; 5 Ifakara Health Institute, Dar es Salaam, United Republic of Tanzania; 6 Department of Global Health and Development, Faculty of Public Health and Policy, London School of Hygiene & Tropical Medicine, London, UK; 7 KEMRI-Wellcome Trust Research Programme Nairobi, Nairobi, Kenya; 8 Department of Paediatrics & Child Health, University of Nairobi, Nairobi, Kenya; 9 Emergency Medicine Department, Muhimbili University of Health and Allied Sciences, Dar es Salaam, United Republic of Tanzania; 10 The George Institute for Global Health India, New Delhi, India; 11 WHO Collaborating Centre for Research in Surgical Care Delivery in LMICs, BARC Hospital, Mumbai, India; 12 Ministry of Health, Community Development, Gender, Elderly and Children, Dodoma, United Republic of Tanzania; 13 Departments of Surgery and Critical Care Medicine, University of Toronto, Toronto, Ontario, Canada; 14 Malawi-Liverpool-Wellcome Trust Clinical Research Programme, Blantyre, Malawi; 15 Department of Disease Control, Faculty of Infectious and Tropical Disease, London School of Hygiene & Tropical Medicine, London, UK; 16 Department of Anaesthesia and Intensive Care, Queen Elizabeth Central Hospital, Blantyre, Malawi; 17 School of Public Health and Family Medicine, College of Medicine, Blantyre, Malawi; 18 Division of Emergency Medicine, University of Cape Town, Cape Town, South Africa; 19 Department of Health Sciences, The Red Cross University College, Huddinge, Sweden; 20 Department of Clinical Research, London School of Hygiene & Tropical Medicine, London, UK

**Keywords:** COVID-19, health systems, health policy, health services research, surgery

## Abstract

**Background:**

Globally, critical illness results in millions of deaths every year. Although many of these deaths are potentially preventable, the basic, life-saving care of critically ill patients are often overlooked in health systems. Essential Emergency and Critical Care (EECC) has been devised as the care that should be provided to all critically ill patients in all hospitals in the world. EECC includes the effective care of low cost and low complexity for the identification and treatment of critically ill patients across all medical specialties. This study aimed to specify the content of EECC and additionally, given the surge of critical illness in the ongoing pandemic, the essential diagnosis-specific care for critically ill patients with COVID-19.

**Methods:**

In a Delphi process, consensus (>90% agreement) was sought from a diverse panel of global clinical experts. The panel iteratively rated proposed treatments and actions based on previous guidelines and the WHO/ICRC’s Basic Emergency Care. The output from the Delphi was adapted iteratively with specialist reviewers into a coherent and feasible package of clinical processes plus a list of hospital readiness requirements.

**Results:**

The 269 experts in the Delphi panel had clinical experience in different acute medical specialties from 59 countries and from all resource settings. The agreed EECC package contains 40 clinical processes and 67 requirements, plus additions specific for COVID-19.

**Conclusion:**

The study has specified the content of care that should be provided to all critically ill patients. Implementing EECC could be an effective strategy for policy makers to reduce preventable deaths worldwide.

**Key questions**

**What is already known?**

- Critical illness is common throughout the world and COVID-19 has caused a global surge of critically ill patients.
- There are large gaps in the quality of care for critically ill patients, especially in low-staffed and low-resourced settings, and mortality rates are high.
- Essential Emergency and Critical Care (EECC) is the effective lifesaving care of low-cost and low-complexity that all critically ill patients should receive in all wards in all hospitals in the world.

**What are the new findings?**

- The clinical processes that comprise EECC and the essential care of critically ill patients with COVID-19 have been specified in a large consensus among clinical experts worldwide.
- The resource requirements for hospitals to be ready to provide this care has been described.

**What do the new findings imply?**

- The findings can be used across medical specialties in hospitals worldwide to prioritise and implement essential care for reducing preventable deaths.
- Inclusion of the EEEC processes could increase the impact of pandemic preparedness and response programmes and policies for health systems strengthening.

## Introduction

Critical illness, when defined as a state of ill health with vital organ dysfunction and a high risk of imminent death, is common in hospitals throughout the world.^1–6^ It is the most severe form of acute illness due to any underlying condition and results in millions of deaths globally every year.^1 5^ The COVID-19 pandemic has led to increased morbidity and mortality with a surge in critical illness worldwide.^7–9^

Many of the deaths due to critical illness are potentially preventable.^10–12^ In critical illness, the patient’s airway, breathing or circulation may become compromised, and early identification of the problem and timely care can be lifesaving. Unfortunately, this care is frequently a neglected part of healthcare. The basic, life-saving clinical processes may be overlooked in specialised care^12^ and in settings of both high^13–15^ and low resources.^16–18^ In hospitals all over the world, guidelines, equipment and routines focusing on the care of critically ill patients, are often missing for adult^19^ and paediatric patients,^11^ in emergency units,^20^ in wards^21^ and in intensive care units.^22^ Improving the way healthcare manages critical illness could save many lives.^11 23 24^

To improve outcomes for critically ill patients by means that are feasible to deliver in all hospital wards and settings, the Essential Emergency and Critical Care (EECC) concept was devised.^25^ EECC is defined as the care that should be provided to all critically ill patients of all ages in all hospitals in the world. It is distinguished by three principles. First, priority to those with the most urgent clinical need, including both early identification and timely care. Second, provision of the life-saving treatments that support and stabilise failing vital organ functions. And third, a focus on effective care of low cost and low complexity.

The clinical processes that comprise the essential care of critically ill patients, and the resources required for those processes have not previously been specified. As critically ill patients can be suffering from any underlying condition, EECC is conceptualised to be integrated into all acute clinical specialties. We therefore sought consensus among a diverse group of global clinical experts with the aim of specifying the content of EECC. An additional aim, given the ongoing pandemic, was consensus around the essential diagnosis-specific care for critically ill patients with COVID-19.

## Methods

**Figure 1:**
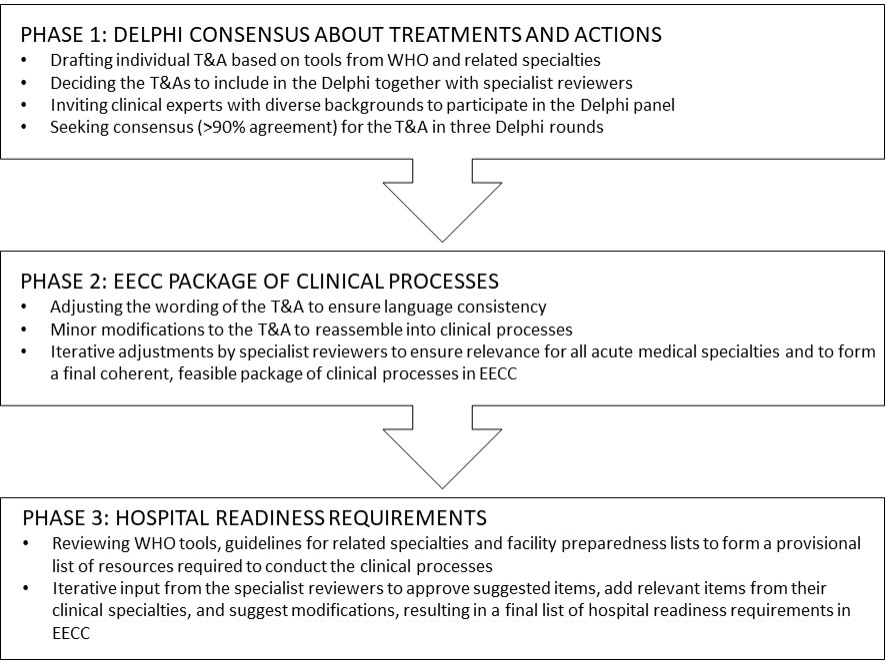
The study process. EECC, Essential Emergency and Critical Care; T&A, Treatment and actions.

The study used three phases (figure 1). First, a consensus was sought about the treatments and actions (T&A) in EECC using a modified Delphi technique.^26^ Second, the output from the Delphi was adjusted into a coherent, user-friendly and feasible package of clinical processes. And third, a list of requirements for hospitals to be ready to provide the care was developed.

### Phase I

An online, three-round modified Delphi process was conducted in November and December 2020. The Delphi method uses anonymous responses from an expert panel to iteratively posed questions and controlled feedback to reach consensus on the topic of interest.^26^ A Delphi process was chosen for this study as EECC is new, its content has not been previously specified and a large group of diverse experts was required.

To be part of the panel, experts needed to have clinical experience of caring for critically ill patients. To ensure the involvement of a diverse range of experts, it was decided that at least 50% of the invitations to participate in the panel should be sent to experts with substantial experience working in low-income and middle-income countries, and there should be a balance between clinical experience (work in general wards, emergency units, intensive care units); specialty (paediatrics, obstetrics, medicine, surgery, intensive care, anaesthesia and emergency care); profession (doctors, nurses, other health professionals); location and gender. A list of potential participants was made from a mapping of stakeholders, the literature across all acute medical specialties, the researchers’ networks and additional purposive and snowball sampling for under-represented groups. Additionally, a link to a screening survey was sent to global professional networks, specialist societies and on social media to identify further potential participants. A total of 895 experts were invited to participate, and those who accepted provided written informed consent.

EECC consists of clinical processes of care. To enable rating by the Delphi panel, clinical processes were disassembled into individual T&A. The T&A concern the identification of critical illness; care of critical illness, and the diagnosis-specific care of critically ill patients with COVID-19. To be included, all T&A were required to meet two a priori defined criteria: *effectiveness* ^i^ and *feasibility.* ^i^ Additionally, *universality* ^i^ was required for the identification and care of critical illness and *relevance* ^i^ was required for the diagnosis-specific care of critically ill patients with COVID-19. A draft list of potential T&A was developed based on clinical guidelines and tools from related specialties^27–38^ and aligned with the WHO/International Committee of the Red Cross’s (ICRC) Basic Emergency Care.^39^ The draft list was revised by specialist reviewers—a group of senior clinicians, researchers and policy makers, with expertise in paediatrics, medicine, emergency medicine, anaesthesia and intensive care, critical care nursing, obstetrics and gynaecology, and surgery.

Three Delphi rounds were deemed sufficient to address the aim while avoiding attrition and poor response rates. A 4-point Likert scale (strongly disagree, disagree, agree, strongly agree) with a ‘do-not know’ option was used for the panel to rate their opinion about the inclusion of each T&A in EECC.^40–42^ Consensus was achieved when more than 90% of respondents selected ‘agree’ or ‘strongly agree’, excluding ‘don’t know’ responses. The experts were able to provide free-text comments, which were analysed to identify appropriate, relevant changes to the wording of T&A for clarity of understanding, and to identify newly proposed T&A. After the first round, newly proposed T&A that fulfilled the EECC criteria for potential inclusion were revised after input from the specialist reviewers and included for assessment by the panel. T&A that did not reach consensus in the previous round were presented for reassessment in rounds two and three, together with a visual representation of the spread of previous responses.

As the Delphi panel was diverse, it was considered that there may be different opinions about the inclusion of T&As between experts with particular a priori defined characteristics. These subgroups of experts were those with work experience in a low-income country or not; those who are doctors or not; those with clinical experience in emergency care and those without; and those with clinical experience in intensive care and those without. The levels of agreement in each subgroup were assessed and presented for all the T&As that reached consensus.

### Phase II

After the Delphi, slight adjustments were made to the wording of the T&A that had reached consensus to ensure language consistency. The T&A were reassembled back into clinical processes to increase overall coherence and feasibility of the EECC package, with the goal of user-friendliness for health system implementation and quality improvement work. The adjustments were done in an iterative process with the same specialist reviewers as in Phase I to ensure relevance for all acute medical specialties. The final package of clinical processes was organised into those relevant for identification, for care and general processes.

### Phase III

A provisional list of hospital readiness requirements for the provision of the clinical processes were developed using existing WHO tools, guidelines for related specialties, facility preparedness lists^29 32 34 35 37–39 43 44^ and the experience and knowledge of the study team. The specialist reviewers provided iterative input into the provisional list, approving suggested items, adding relevant items from their clinical specialties and suggesting modifications. Based on previous work and following consultation with health economists and procurement experts, the final list of requirements was agreed and arranged into eight categories: equipment, consumables, drugs, human resources, training, routines, guidelines and infrastructure.

### Patient and public involvement

Patients and the public were not involved in the design and conduct of the research.

## Results

### Phase I

**Figure 2:**
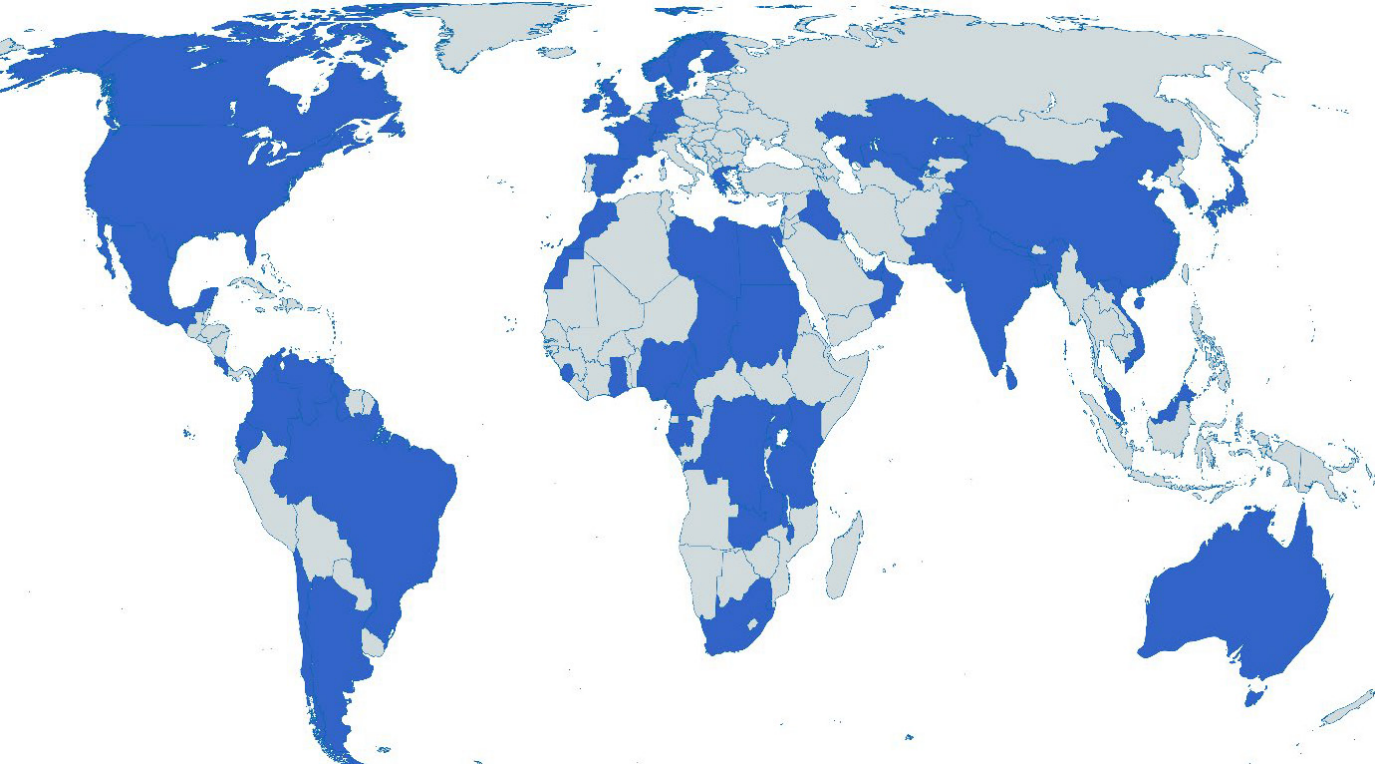
The expert panel locations. Created with mapchart.net. Disclaimer: the depictions of boundaries are not warranted to be error free.

Of the 895 invited experts, 269 participated in the first round of the Delphi when the majority of the decisions were made (30% response rate). In round 2, 228 experts participated (85% of those in round 1) and round 3 included 194 experts (85% of those in round 2). The panel comprised experts from diverse resource settings, clinical settings, specialties and professions (table 1). The panel included experts from 59 countries (figure 2) and 38% were female.

**Table 1:** The characteristics of the expert panel in the Delphi (first round)

	Number of experts (N=269)	Proportion of experts (%)*
Resource setting*		
High-income country	139	52
Middle-income country	115	43
Low-income country	177	66
Do not know	2	1
Clinical setting*		
General ward	153	57
Emergency unit	179	67
High dependency unit	153	57
Intensive care unit	232	86
Operating theatre	102	38
Other	15	6
Specialty*		
Emergency care	93	35
Intensive care	190	71
Anaesthesia	59	22
Medicine	39	15
Surgery	20	7
Paediatrics	47	17
Obstetrics/gynaecology	13	5
Other	25	9
Profession*		
Doctor	212	79
Nurse	40	15
Midwife	6	2
Clinical officer	9	3
Other	17	6
Gender*		
Female	102	38
Male	165	62

*As the experts were asked to select all that apply, the sum of the percentages may exceed 100.

Of the 57 T&A for EECC in round 1, consensus was reached for 49. In round 2, 29 newly proposed T&A were added to the eight remaining from round 1, of which two had been reworded for clarity. Out of these 37, consensus was reached for 17. The remaining 20, of which another two had been reworded for clarity, were included in round 3. Consensus was reached for nine of the final 20 T&A. In total, consensus was reached for 75 out of 86 proposed T&A, including 54 of the original 57 (online supplemental table 1).

10.1136/bmjgh-2021-006585.supp1 Supplementary data

Of the seven T&A for the essential diagnosis-specific care of critically ill COVID-19 in round 1, all reached consensus for inclusion. In round 2, two newly proposed T&A were added. Neither of these reached consensus in round 2 or round 3.

Analyses of participant subgroups did not reveal substantial divergence from the overall results. For the T&A that reached 90% agreement in the panel, agreement was not below 80% in any subgroup (online supplemental tables 2–4).

### Phase II and III

After the Delphi, the T&A that had reached consensus were reassembled into a final user-friendly and feasible package of EECC containing 40 clinical processes—30 identification and care processes and 10 general processes (table 2). All T&A for the care of critical illness were included, with some rewording and reordering. Eleven T&A for the identification of critical illness were not included, so that the package could be feasible for triage in all hospitals, and were added as an addendum (outside the remit of EECC), in order to underscore their importance in settings where staff have sufficient time and expertise.

**Table 2:** The clinical processes of Essential Emergency and Critical Care

**Identification of critical illness** *Critical illness is identified as soon as possible so timely care can be provided*	
1. The hospital uses vital signs-based triage to identify critical illness	
1.1 Triage/identification of critical illness includes the use of these vital signs	
1.1.1 Pulse rate 1.1.2 Blood pressure 1.1.3 Respiratory rate 1.1.4 Oxygen saturation (SpO_2_) 1.1.5 Temperature 1.1.6 Level of consciousness (eg, ‘AVPU’, ‘ACVPU’ or Glasgow Coma Scale) 1.1.7 Presence of abnormal airway sounds heard from the bedside (eg, snoring, gurgling, stridor) 1.1.8 The overall condition of the patient (health worker’s concern that the patient is critically ill)	
1.2 Triage/identification of critical illness is conducted at these times	
1.2.1 When a patient arrives at hospital seeking acute care 1.2.2 For hospital in-patients, at least every 24 hours, unless otherwise prescribed, with increased frequency for patients who are at risk of becoming critically ill or who are critically ill, and then less frequently again when patients are stabilising 1.2.3 When a health worker, or the patient or guardian, is concerned that a patient may be critically ill 1.2.4 During and after surgery or anaesthesia 1.2.5 During and after transport/transfer of a patient who is critically ill or at risk of becoming critically ill 1.2.6 Following a treatment or action (re-evaluation)	
**Care of critical illness** *Essential care of critical illness is initiated as soon as critical illness is identified and involves these clinical processes when appropriate*	
Airway	2. Placing the patient in the recovery position (lateral position) 3. Age-appropriate airway positioning (eg, chin lift or jaw thrust in adults, neutral position in young children) 4. Removal of any visible foreign body from the mouth or use of age-appropriate chest thrusts/abdominal thrusts/back blows in choking 5. Suction for secretions that are obstructing the airway 6. Insertion of an oropharyngeal (Guedel) airway
Care for a blocked or threatened airway	
Breathing	7. Optimising the patient’s position (eg, sitting-up or prone) 8. Oxygen therapy using nasal prongs, facemask or mask with a reservoir bag (non-rebreathing mask) 9. Bag-valve-mask ventilation in threatened or manifest respiratory arrest
Care for hypoxia or respiratory distress	
Circulation	10. Optimising the patient position (eg, lying flat, head-down, raised-legs, lateral tilt in pregnancy) 11. Compression and elevation to stop bleeding 12. Appropriate bolus of intravenous fluid 13. Oral rehydration solution or other appropriate oral fluids for dehydration without shock 14. Intramuscular epinephrine for anaphylaxis 15. Uterine massage and/or oxytocin when indicated
Care for a threatened circulation or shock	
Reduced conscious level	16. Treating an unconscious patient as having a threatened airway (eg, recovery position, etc) 17. Dextrose (intravenous or buccal) in unconsciousness or seizures unless bedside blood glucose testing rules out hypoglycaemia or there is a clear alternative cause 18. Protecting patients with a seizure from harm 19. Quick-acting antiseizure medication (eg, intravenous/rectal diazepam or magnesium sulphate in pregnancy/post partum) 20. Cooling in severe hyperthermia with a reduced level of consciousness
Care for a reduced level of consciousness	
Other care in EECC	21. Insertion of an intravenous cannula when critical illness is identified 22. Insertion of an intraosseous cannula, if indicated, if an intravenous cannula is not possible 23. Stabilising the cervical spine in possible cervical spine injury 24. Appropriate antibiotics for sepsis 25. Treatment of pain and anxiety (eg, with needs-based psychological support, medication) 26. Keeping the patient warm using blankets and other means (including skin-to-skin care for babies) 27. Feeding (including breast feeding for babies), nasogastric feeding and dextrose for nutrition and to avoid hypoglycaemia 28. Prevention of delirium (eg, sleep hygiene, provision of the patient’s glasses or hearing aid) 29. Regular turning of immobilised patients 30. Mobilising the patient as early as possible
Other immediate or ongoing care of critical illness	
**General processes** *Care is provided according to these general processes*	
31. Assistance from additional or senior staff is sought when a critically ill patient is identified 32. Essential Emergency and Critical Care (EECC) is respectful and patient-centred 33. EECC is provided without considering the patient’s ability to pay 34. Critically ill patients are cared-for in locations that facilitate observation and care (eg, designated beds, a bay or a unit for critically ill patients) 35. Infection, prevention and control measures are used including hand hygiene and separation of patients with a suspected or confirmed contagious disease from those without 36. Communication is clear, including: Within the care team when a patient is identified as critically ill (eg, verbal communication, at staff handovers, visible colour-coding) Within the care team about the planned EECC (eg, continue oxygen therapy, give intravenous fluids) Documentation in the patient notes about the vital signs, when critical illness has been identified and the treatments and actions conducted Effective and respectful communication with the patient and family 37. If there is poor response to treatment, or if the patient deteriorates, other indicated EECC clinical processes are used 38. Clinical processes are discontinued that are no longer indicated (eg, if a patient improves or if they are deemed to no longer be in the patient’s best interest) 39. It is recognised when EECC alone is not sufficient to manage the critical illness 40. EECC is integrated with care that is outside the scope of EECC (eg, the need for prompt investigations, definitive treatment of underlying conditions including following disease-specific best-practice guidelines, end-of-life care, referral)	
**Addendum: extended identification of critical illness**	
To maintain feasibility of the EECC package, only a limited number of signs for the identification of critical illness are included. However, if time and expertise allow, there are additional signs that are not part of EECC that aid the identification of critical illness: Presence of respiratory distress (eg, unable to complete sentences; accessory muscle use; chest recessions; grunting or head nodding) Cyanosis Capillary refill time Cold or warm extremities Presence of severe dehydration (eg, decreased skin turgor; dry mucous membranes; sunken fontanelle) Confused, agitated or disoriented mental state Presence of prostration or lethargy Presence of a generalised seizure Inability to stand or walk without help Inability to breast feed or feed in a young child Presence of severe acute malnutrition	

AVPU Alert, Voice, Pain, Unresponsive; ACVPU Alert, Confusion, Voice, Pain, Unresponsive

The list of hospital readiness requirements for EECC contained 67 items, (14 for identification and 53 for essential care) (table 3).

**Table 3:** The hospital readiness requirements for Essential Emergency and Critical Care

**Identification of critical illness** *The following items are required for a hospital to be ready for the identification of critically ill patients*	
**Category**	**Item**
1.1. Equipment	1.1.1 Clock with secondhand 1.1.2 Pulse oximeter and probe 1.1.3 Blood pressure measuring equipment (eg, sphygmomanometer with a stethoscope) 1.1.4 Blood pressure cuffs of different paediatric and adult sizes 1.1.5 Light source (lamp or flashlight) 1.1.6 Thermometer
1.2 Consumables	1.2.1 Soap or hand disinfectant 1.2.2 Examination gloves
1.3 Drugs	None
1.4 Human resources	1.4.1 Health workers with the ability to identify critical illness 24 hours/day
1.5 Training	1.5.1 The health workers are trained in the identification of critical illness
1.6 Routines	1.6.1 Routines for the identification of critical illness
1.7 Guidelines	1.7.1 Guidelines for the identification of critical illness
1.8 Infrastructure	1.8.1 Designated triage area (area for the identification of critical illness) in the Out-Patient Department or Emergency Unit (area of the hospital where patients arrive) 1.8.2 Running water
**Care of critical illness** *The following items are required for a hospital to be ready to provide the care of critically ill patients*	
2.1 Equipment	2.1.1 Suction machine (electric or manual) 2.1.3 Oxygen supply 24 hours/day (cylinder, concentrator (with electricity supply) or piped oxygen) 2.1.4 Flow meter (if using cylinder or piped oxygen) 2.1.5 Leak-free connectors from oxygen source to tubing 2.1.6 Bag valve mask (resuscitator)—neonatal, paediatric and adult sizes 2.1.7 Sharps disposal container 2.1.8 External heat source
2.2 Consumables	2.2.1 Suction catheters of paediatric and adult sizes 2.2.2 Guedel airways of paediatric and adult sizes 2.2.3 Pillows 2.2.4 Oxygen tubing 2.2.5 Oxygen nasal prongs 2.2.6 Oxygen face masks of paediatric and adult sizes 2.2.7 Oxygen face masks with reservoir bags of paediatric and adult sizes 2.2.8 Masks for bag valve mask (resuscitator)—neonatal, paediatric and adult sizes 2.2.9 Compression bandages 2.2.10 Plasters or tape 2.2.11 Gauze 2.2.12 Intravenous cannulas of paediatric and adult sizes 2.2.13 Intravenous giving sets 2.2.14 Skin disinfectant for cannulation 2.2.15 Syringes 2.2.16 Nutrition 2.2.17 Nasogastric tubes 2.2.18 Lubricant for nasogastric tube insertion 2.2.19 Intramuscular needles 2.2.20 Intraosseous cannulas of different sizes 2.2.21 Blankets 2.2.22 Facemasks for infection prevention and control 2.2.23 Aprons or gowns 2.2.24 Charts/notes for documentation 2.2.25 Pens
2.3 Drugs	2.3.1 Oral rehydration solution 2.3.2 Intravenous crystalloid fluids (eg, normal saline or Ringer’s Lactate) 2.3.3 Intravenous dextrose fluid (eg, 5%, 10% or 50%) 2.3.4 Oxytocin 2.3.5 Epinephrine 2.3.6 Appropriate antibiotics 2.3.7 Diazepam 2.3.8 Magnesium sulphate 2.3.9 Paracetamol 2.3.10 Local anaesthetic (eg, 2% lignocaine) (eg, for intraosseous cannulation)
2.4 Human resources	2.4.1 Health workers with the ability to care for critically ill patients 24 hours/day 2.4.2 Senior health worker who can be called to assist with the care of critically ill patients 24 hour/day
2.5 Training	2.5.1 The health workers are trained in the care of critically ill patients
2.6 Routines	2.6.1 Routines for managing critically ill patients 2.6.2 Routine for the provision of EECC without taking into account patients’ ability to pay 2.6.3 Routines for who and how to call to seek senior help 24 hours/day, 7 days/week 2.6.4 Routines for integrating EECC with other care including the definitive care of the underlying condition (eg, use of condition-specific guidelines)
2.7 Guidelines	2.7.1 Guidelines for the essential care of critically ill patients
2.8 Infrastructure	2.8.1 Designated space for the care of critically ill patients (eg, a bay, ward, high dependency unit) 2.8.2 Areas for separating and managing patients with a suspected or confirmed contagious disease from those without

The essential diagnosis-specific care of critically ill patients with COVID-19 consisted of an additional seven clinical processes and nine hospital readiness requirements (table 4).

**Table 4:** The essential diagnosis-specific care for critically ill patients with COVID-19

**Clinical processes**	
The Essential Emergency and Critical Care (EECC) clinical processes as specified for all critical illnesses Personal protective equipment (PPE) that is appropriate for COVID-19 as part of infection, prevention and control Monitoring oxygen saturation using pulse oximetry at least every 6 hours, unless otherwise prescribed Intermittent prone positioning Low molecular weight heparin or other anticoagulant Corticosteroid Antibiotics in patients with suspected bacterial superinfection	
**Hospital readiness requirements**	
Critically ill patients with COVID-19 require the same hospital readiness for EECC as other critically ill patients. For the provision of the essential diagnosis specific care of critically ill patients with COVID-19, the following additional items are required	
**Category**	**Item**
		3.1 Equipment	None
3.2 Consumables	3.2.1 Facemasks appropriate for COVID-19 (eg, N95) 3.2.2 Eye protection or face shields
3.3 Drugs	3.3.1 Low-molecular weight heparin (eg, enoxaparin or dalteparin) or other anticoagulant 3.3.2 Corticosteroid (eg, dexamethasone)
3.4 Human resource	3.4.1 Health workers with the ability to care for critically ill patients with COVID-19 24 hours/day
3.5 Training	3.5.1 The health workers are trained in essential care of critically ill patients with COVID-19
3.6 Routines	3.6.1 Routines for care of critically ill patients with COVID-19
3.7 Guidelines	3.7.1 Guidelines for essential care of critically ill patients with COVID-19
3.8 Infrastructure	3.8.1 Areas for separating and managing patients with suspected or confirmed COVID-19 from those without

## Discussion

We have specified the content of EECC based on consensus among global clinical experts. While the EECC approach is new, the included clinical processes are commonly used in the care of sick patients and can be seen in WHO publications and specialist society standards and guidelines.^29 32–35 38 39 45^ The contribution of this study is the specification of a baseline bundle of care interventions that should be provided when needed to all critically ill patients in all hospitals in the world. This marks a break from previous guidelines that tend to be specialty-specific, condition-specific or location-specific, or that specify care that may be too complex and costly to provide in all hospital settings.

### The EECC approach

EECC is an approach that supports priority-setting in health systems. In this regard, it has parallels to the approaches used in the WHO’s Essential Medicines List,^37^ Interagency Integrated Triage Tool,^29^ Emergency Triage and Treatment for Children^32^ and Universal Health Coverage.^36^ EECC emphasises the identification and care of the critically ill, and the provision of the life-saving supportive care that is of low cost and of low complexity.^25^ EECC can be seen as a unifying concept for such aspects of patient management found in WHO and specialist guidelines, triage, early warning systems and rapid response teams.^28 29 39 46 47^ To maintain focus on life-saving supportive care and to be useful across all specialties, EECC does not include the definitive care of the underlying diagnoses. Instead, EECC is intended to complement specialty-based care and existing guidelines and does not aim to include all the care a patient needs—as well as EECC, patients should receive diagnostics, definitive and symptomatic care of their condition, additional nursing care, and if available, higher levels of emergency and critical care. EECC seeks to bridge the quality gap that is commonly found between the current care of critical illness and best-practice guidelines.^12 48 49^ To ensure feasibility in settings with restricted human resources, EECC is designed to enable task-sharing between health professionals.^50^ It should be noted that not all the EECC clinical processes will be needed in the care of every critically ill patient—they should be seen as essential ‘tools in the tool-box’ for health workers to use when required. To operationalise the EECC approach, it is intended that the content specified here is used to develop tools for quality monitoring, teaching and integration into other guidelines and recommendations.

### EECC complements the current healthcare organisation

The basic clinical processes specified in EECC have been overlooked in healthcare.^11 13–15 18 20 51 52^ In UK hospitals, half of the patients received substandard basic vital organ support prior to intensive care and 31% of preventable deaths were associated with absent clinical monitoring.^13 14^ In Malawi, 89% of adult hypoxic patients and 75% of children dying from pneumonia in hospital did not receive oxygen.^17 18^ The usual organisational set-up of health services may be one underlying reason for this. Specialist units with a primary function of delivering the definitive management for one disease group may under-estimate the effort needed to maintain core processes and competences in the supportive management of critically ill patients. Innovative and specialised treatments and technologies may become preferred to those that are basic and long-standing.^53^ By targeting a feasible, lowest baseline quality for critically ill patients throughout hospital settings, EECC provides a complimentary approach to the current organisation that safeguards the provision of basic life-saving actions, enhancing the impact of hospital care for all acute conditions.

### EECC in the COVID-19 pandemic

EECC has added importance in a situation causing a substantial amount of severe disease and the Delphi panel agreed that EECC should be part of the care of critically ill patients with COVID-19. In addition, the agreed essential diagnostic-specific care for COVID-19 can assist in decisions about the priorities of care when the pandemic threatens to overwhelm available resources. All of the COVID-19 specific processes are well established and are included in the WHO COVID-19 clinical management guidance.^30^ The WHO guidance, and others,^54^ additionally include recommendations for advanced critical care (such as mechanical ventilation, vasopressors and extracorporeal oxygenation), which may be difficult to rapidly scale-up in settings of low resources. Advanced critical care can be necessary to save the lives of some patients, but has a high cost per recovery and risks diverting scarce resources to a few individuals.^55–60^ Fortunately, the focus has shifted in the global pandemic response from advanced critical care towards securing basic oxygen delivery systems^61 62^ underscored by statements from the WHO and other partners.^63 64^ The impact of this shift, in and beyond the pandemic, could be even greater if the necessary processes for the effective use of oxygen and other care specified in EECC were included in the scale-up.

### Strengths and limitations

Our use of a consensus method with a large expert panel from diverse clinical and resource settings, specialties and geographical locations gives the specified content legitimacy. The high response rate for this type of study during an ongoing pandemic illustrates the interest that experts had in the project’s aims. The high level of consensus (>90%) for the included clinical processes promotes confidence in the final package. However, the Delphi method does have limitations. It is expert-opinion based and is limited by the make-up of the panel. Only English language speakers were included, experts were not included from all countries and the expedited timeline of the project due to the need for results that could impact the global response to the COVID-19 pandemic may have excluded experts who could have provided additional input. The initial content presented to the panel was aligned with WHO initiatives,^39^ and developed by a diverse specialist team, but the possibility remains that alternative methods would have led to a different output. The study did not address the underlying evidence-base for the included clinical processes, the impact, or the potential opportunity costs of increasing the coverage of EECC in hospitals—such system-wide effects warrant careful evaluation during EECC implementation. It should be noted that, while policy makers were involved throughout the process, the EECC content has not been ratified by the WHO or governmental ministries of health—the method has been primarily scientific. The findings should be seen as the first version of the EECC content, as recommended by global clinicians and researchers, one that could be incorporated into WHO and other global and national programmes and that should subsequently be improved and updated as new knowledge arises.

### Implications

Implementation of EECC could be an effective strategy as part of the current calls to save lives through improved quality of care in health systems^65^—a ‘low-hanging fruit’. Critically ill patients have high mortality rates in all hospital settings, especially where trained staff or resources are limited, and even small improvements in outcomes would have a large impact. EECC has a vital role in the ongoing COVID-19 pandemic, for the care of the surge of critically ill patients and for optimising the impact of the efforts to scale-up oxygen. Policy makers at global, national and regional levels aiming to reduce preventable deaths should focus on improved coverage of EECC and inclusion of EECC as part of universal health coverage.^36^

## Conclusion

The content of EECC—and the essential care of critically ill patients with COVID-19—has been specified using an inclusive global consensus. The content consists of effective, low-cost and low-complexity life-saving care that is still frequently overlooked. The time has come to ensure that all patients in the world receive this care.

## Data Availability

All data relevant to the study are included in the article or uploaded as supplementary information. Access to anonymised data may be provided to researchers after provision of a study protocol and justification to the corresponding author.

## Footnotes

Effectiveness: established or proven to be safe and to reduce mortality (compression to stop bleeding is effective; treating with leech therapy is not). *Feasibility*: low-cost and low complexity. Possible to provide in a low-staffed, low-resourced setting without the immediate presence of a doctor (placing a comatose patient in the recovery position (lateral position) is feasible; continuous haemodialysis is not). *Universality*: supports vital organ function rather than being the definitive care of a diagnosis (intravenous fluids for shock are universal; thrombolytic therapy is not). *Relevance*: established or proven to be a treatment for COVID-19.
