# Supplementary material for: Essential Emergency and Critical Care: a consensus among global clinical experts
Source: BMJ Glob Health. 2021 Sep 21;6(9):e006585. doi: 10.1136/bmjgh-2021-006585 (PMC8458367; doi:10.1136/bmjgh-2021-006585)
Supplement: Supplementary data [file bmjgh-2021-006585supp001.pdf]

BMJ Publishing Group Limited (BMJ) disclaims all liability and responsibility arising from any reliance placed on this supplemental material which has been supplied by the author(s)

Supplementary material

Contents

Supplementary Table 1. Delphi Results 2
Supplementary Table 2 Subgroup analysis Round One 11
Supplementary Table 3 Subgroup analysis Round Two 14
Supplementary Table 4: Subgroup analysis Round Three 15

Schell CO, *et al. BMJ Global Health* 2022; 6:e006585. doi: 10.1136/bmjgh-2021-006585

Supplementary Table 1. Delphi Results

Level of agreement among Delphi experts in each round for the treatments and actions. Agreement is defined as the proportion of experts that rated "strongly agree" or "agree" out of those who provided a rating. Consensus is defined as ≥90% agreement (green), and non-consensus is <90% (orange).
EECC; Essential Emergency and Critical care. AVPU; Alert, Voice, Pain, Unresponsive.

Treatments and Actions	Round 1 N=269 Agreement (%)	Round 2 N=228 Agreement (%)	Round 3 N=194 Agreement (%)
1. Identification of critical illness in EECC includes evaluating a limited number of physiological signs. The physiological signs used in EECC should include:			
The overall condition of the patient (concern that the patient is critically ill)	96.6		
Presence of abnormal airway sounds (eg. snoring, gurgling, stridor)	97.0		
Respiratory rate	99.3		
Oxygen saturation (SpO2)	95.9		
Pulse rate	99.6		
Blood pressure	95.9		
Level of consciousness (eg. "AVPU" or Glasgow Coma Scale)	99.3		
Temperature	88.0	85.9	93.3
Capillary refill time		84.0	94.8
Cold or warm extremities		80.9	83.0
Cyanosis		82.3	87.5
Confused, agitated or disoriented mental state		89.9	94.3
Presence of prostration or lethargy		77.5	83.4
Inability to stand or walk without help		63.1	64.8
Presence of a generalized seizure		82.1	91.1

Schell CO, *et al. BMJ Global Health* 2022; 6:e006585. doi: 10.1136/bmjgh-2021-006585

Presence of respiratory distress (eg unable to complete sentences; accessory muscle use; chest recessions; grunting or head nodding)		93.4	
Presence of severe dehydration (eg decreased skin turgor; dry mucous membranes; sunken fontanelle)		91.2	
Inability to breastfeed or feed in a young child		86.0	92.9
Presence of severe acute malnutrition		72.8	83.8
2. An evaluation of physiological signs should be conducted at these times:			
When a patient arrives at hospital seeking acute care	100		
When a health worker is concerned that a patient may be critically ill	98.9		
For hospital in-patients, at least every 24 hours, unless otherwise prescribed (Note: can be done more frequently)	86.9	89.4	96.4
More frequently for patients who are at risk of becoming critically ill or who are critically ill (eg. every 12hrs, every 6 hrs ... etc)	99.6		
Less frequently for patients who have improved and are now stable (eg ...every 6hrs, every 12hrs, every 24hrs)	84.4	95.6	
Following a treatment or action (re-evaluation)	98.9		
When a patient, family member or guardian is concerned that the patient may be critically ill		88.6	94.8
During and after transport/transfer of a patient who is critically ill or at risk of becoming critically ill		98.3	
During and after surgery or anaesthesia		95.6	

Schell CO, *et al. BMJ Global Health* 2022; 6:e006585. doi: 10.1136/bmjgh-2021-006585

Treatments and Actions	Round 1 N=269 Agreement (%)	Round 2 N=228 Agreement (%)	Round 3 N=194 Agreement (%)
3. Communication about the patient's physiological signs in EECC should include:			
Documentation of the vital signs in the patient notes	99.6		
Documentation in the patient notes when critical illness has been identified	98.5		
Clear communication within the care team that a patient is critically ill (eg. verbal communication, at staff handovers, visible colour-coding)	99.6		
4. EECC includes treatment & actions when a patient has a blocked or threatened airway. The treatments & actions for a blocked or threatened airway should include:			
Recovery position (lateral position)	91.3		
Age-appropriate airway positioning (eg chin lift or jaw thrust in adults, neutral position in young children)	99.6		
Removal of any visible foreign body from the mouth	98.1		
Suction for secretions that are obstructing the airway	97.8		
Oro-pharyngeal (Guedel) airway	92.1		
Naso-pharyngeal airway	85.4	84.4	83.7
Age-appropriate chest thrusts/ abdominal thrusts/ back blows in choking	94.3		

5. EEcc includes treatments & actions when a patient has respiratory distress or hypoxia. The treatments & actions for respiratory distress or hypoxia should include:			
Optimize patient position (eg. sitting-up or prone)	99.3		
Oxygen therapy using nasal prongs	90.0	91.7	
Oxygen therapy using facemask	97.4		
Oxygen therapy using a mask with a reservoir bag (non re-breathing mask)	95.8		
Breathing exercises (eg. deep breaths, coughing, changing position, expiration against mild resistance)	78.5	76.4	72.1
Inhaled bronchodilators (eg salbutamol)	82.8	84.0	86.5
Bag-valve-mask ventilation for newborns	97.9		
Bag-valve-mask ventilation for children		91.3	
Bag-valve-mask ventilation for adults		86.7	96.8

**Treatments and Actions**	**Round 1 N=269 Agreement (%)**	**Round 2 N=228 Agreement (%)**	**Round 3 N=194 Agreement (%)**
6. EECC includes treatments & actions when a patient has a threatened circulation or shock. The treatments & actions for a threatened circulation or shock should include:			
Optimise patient position (eg. lying flat, head-down, raised-legs, lateral tilt in pregnancy)	99.3		
Compression and elevation to stop bleeding	99.6		
Uterine massage when indicated	97.9		
Appropriate bolus of intravenous fluid	98.5		
Oral rehydration solution or other appropriate oral fluids for dehydration without shock	81.0	94.3	
Intramuscular adrenaline for anaphylaxis		90.6	
Blood transfusion		75.5	85.3
Tourniquet in severe limb bleeding†		74.2	88.2
Appropriate antibiotics for sepsis		97.4	
Oxytocin when indicated		96.2	
Tranexamic Acid for haemorrhage		79.1	83.1
7. EECC includes treatments & actions when a patient has a reduced level of consciousness. The treatments & actions for a reduced level of consciousness should include:			
Treating an unconscious patient as having a threatened airway	99.3		
Dextrose (iv or buccal) in unconsciousness or seizures unless bedside blood glucose testing rules out hypoglycaemia or there is a clear alternative cause	92.8		
Protecting patients with a seizure from harm	98.1		

Quick-acting anti-seizure medication for prolonged seizures (eg. iv or rectal diazepam)	98.9		
Quick-acting anti-seizure medication for seizures in pregnancy and post-partum (eg. im magnesium sulphate)	99.2		
Cooling (eg. fans, antipyretics) in severe hyperthermia with a reduced level of consciousness.	93.5		

Schell CO, *et al. BMJ Global Health* 2022; 6:e006585. doi: 10.1136/bmjgh-2021-006585

Treatments and Actions	Round 1 N=269 Agreement (%)	Round 2 N=228 Agreement (%)	Round 3 N=194 Agreement (%)
8. EECC includes additional clinical treatments & actions for critical illness. These clinical treatments & actions should be included in EECC:			
Use of other indicated EECC treatments & actions if there is poor response to treatment, or if the patient deteriorates	98.8		
Discontinuation of a treatment or action that is no longer indicated if a patient improves	94.7		
Discontinuation of a treatment or action that has been deemed to no longer be in the patient's best interest	94.7		
Use of blankets and other means (including skin-to-skin care for babies) to keep the patient warm	99.2		
Feeding (including breastfeeding for babies). naso-gastric feeding and dextrose for nutrition and to avoid hypoglycaemia	98.1		
Treatment of pain and anxiety (eg. with needs-based psychological support, medication)	98.9		
Mobilise the patient as early as possible		91.1	
Insertion of an intravenous cannula when critical illness is identified		98.2	
Insertion of an intraosseous cannula, if indicated, if an IV cannula is not possible		90.9	
Regular turning of immobilised patients		94.3	
Cervical spine stabilization in possible cervical spine injury		96.9	

9. EECC includes additional treatments & actions for critical illness. These additional treatments & actions should be included in EECC:			
Infection, prevention and control (IPC) measures including hand hygiene and separation of patients with a suspected or confirmed contagious disease from those without	98.9		
Seeking of assistance from additional or senior staff when a critically ill patient is identified	99.3		
Care for all critically ill patients in locations that facilitate observation and care (eg. designated beds, a bay or a unit for critically ill patients)	99.6		
Respectful and patient-centred care	99.3		
Effective and respectful communication with the patient and family	99.3		
Provision of EECC without considering the patient's ability to pay	97.0		
Clear documentation of all treatments & actions	99.6		
Recognition when EECC is not sufficient to manage the critical illness	98.9		
Integration with care that is outside the scope of EECC (eg. the need for prompt investigations, definitive treatment of underlying conditions, end-of-life care, referral)	98.9		
Clear communication and documentation about the planned EECC (eg continue oxygen therapy, give IV fluids		97.4	
Prevent delirium (eg. sleep hygiene, provision of the patient's glasses or hearing aid)		84.9	91.6

Treatments and Actions	Round 1 N=269 Agreement (%)	Round 2 N=228 Agreement (%)	Round 3 N=194 Agreement (%)
10. The essential care of a critically ill COVID-19 patient should include:			
The EECC treatments & actions as specified for all critical illnesses	98.9		
Personal Protective Equipment (PPE) that is appropriate for COVID-19 as part of Infection, Prevention and Control (IPC)	99.6		
Monitoring oxygen saturation using pulse oximetry at least every 6 hours, unless otherwise prescribed	91.7		
Intermittent prone positioning	90.9		
Low molecular weight heparin or other anticoagulant	92.2		
Corticosteroid	93.8		
Antibiotics in patients with suspected bacterial superinfection	98.5		
Zinc		51.5	42.8
Aspirin		54.5	57.1

Supplementary Table 2 Subgroup analysis Round One

Agreement in the sub-groups of experts for the treatments and actions that reached consensus in Round One. Agreement is defined as the proportion of experts that rated "strongly agree" or "agree" out of those who provided a rating. Consensus is ≥90% agreement.

Reached Consensus in Round 1	Delphi Panel	Intensive Care		Emergency Care		Low Income Country		Doctor	
**Treatments and Actions**		**Intensive Care**	**Non-Intensive Care**	**Emergency Care**	**Non-Emergency Care**	**LICs**	**Non-LICs**	**Doctor**	**Non-doctor**
1.1 The overall condition of the patient (concern that the patient is critically ill)	96.6	96.9	96.2	97.9	96.0	95.5	98.9	96.7	96.5
1.2 Presence of abnormal airway sounds (eg. snoring, gurgling, stridor)	97.0	96.3	98.7	98.9	96.0	98.9	93.5	96.7	98.3
1.3 Respiratory rate	99.3	99.5	98.7	98.9	99.4	99.4	98.9	99.1	100
1.4 Oxygen saturation (SpO2)	95.9	96.3	94.8	93.6	97.1	97.1	93.5	94.8	100
1.5 Pulse rate	99.6	100	98.7	98.9	100	99.4	100	99.5	100
1.6 Blood pressure	95.9	96.3	94.8	95.7	96.0	96.0	95.7	94.8	100
1.7 Level of consciousness (eg. "AVPU" or Glasgow Coma Scale)	99.3	99.5	98.7	98.9	99.4	98.9	100	99.1	100
2.1 When a patient arrives at hospital seeking acute care	100	100	100	100	100	100	100	100	100
2.2 When a health worker is concerned that a patient may be critically ill	98.9	99.0	98.7	98.9	98.9	98.9	98.9	99.1	98.3
2.6 Following a treatment or action (re-evaluation)	98.9	98.9	98.7	98.9	98.9	98.3	100	99.1	98.3
3.1 Documentation of the vital signs in the patient notes	99.6	99.5	100	100	99.4	99.4	100	99.5	100
3.2 Documentation in the patient notes when critical illness has been identified	98.5	98.4	98.7	97.9	98.9	97.7	100	99.1	96.5
3.3 Clear communication within the care team that a patient is critically ill (eg. verbal communication, at staff handovers, visible colour-coding)	99.6	99.5	100	98.9	100	99.4	100	100	98.3
4.1 Recovery position (lateral position)	91.3	91.4	91.0	91.1	91.4	90.4	93.2	92.4	87.3
4.2 Age-appropriate airway positioning (eg chin lift or jaw thrust in adults, neutral position in young children)	99.6	99.5	100	100	99.4	100	98.9	99.5	100
4.3 Removal of any visible foreign body from the mouth	98.1	97.3	100	97.9	98.3	97.7	98.9	97.6	100
4.4 Suction for secretions that are obstructing the airway	97.8	98.4	96.2	97.9	97.7	97.2	98.9	97.2	100
4.5 Oro-pharyngeal (Guedel) airway	92.1	92.1	92.1	87.0	94.8	93.7	89.0	90.9	96.4
4.7 Age-appropriate chest thrusts/ abdominal thrusts/ back blows in choking	94.3	93.1	97.4	94.6	94.2	94.9	93.3	93.8	96.4
5.1 Optimize patient position (eg. sitting-up or prone)	99.3	99.5	98.7	97.9	100	99.4	98.9	99.5	98.3

5.3 Oxygen therapy using facemask	97.4	99.0	93.7	97.9	97.2	97.7	96.7	99.1	91.2
5.4 Oxygen therapy using a mask with a reservoir bag (non re-breathing mask)	95.8	95.8	96.0	94.6	96.5	96.5	94.5	95.2	98.3
5.7 Bag-valve-mask ventilation for newborns	97.9	97.6	98.6	97.7	98.0	98.2	97.4	97.8	98.2
6.1 Optimise patient position (eg. lying flat, head-down, raised-legs, lateral tilt in pregnancy)	99.3	99.0	100	98.9	99.4	98.9	100	99.0	100
6.2 Compression and elevation to stop bleeding	99.6	99.5	100	98.9	100	99.4	100	100	98.3
6.3 Uterine massage when indicated	97.9	98.8	95.6	98.8	97.4	98.2	97.3	97.3	100
6.4 Appropriate bolus of intravenous fluid	98.5	98.4	98.7	97.9	98.9	99.4	96.7	98.1	100
7.1 Treating an unconscious patient as having a threatened airway	99.3	100	97.4	100	98.9	99.4	98.9	99.5	98.3
7.2 Dextrose (iv or buccal) in unconsciousness or seizures unless bedside blood glucose testing rules out hypoglycaemia or there is a clear alternative cause	92.8	92.0	94.7	96.7	90.6	93.6	91.1	92.3	94.6
7.3 Protecting patients with a seizure from harm	98.1	97.9	98.7	98.9	97.7	98.3	97.8	97.6	100
7.4 Quick-acting anti-seizure medication for prolonged seizures (eg. iv or rectal diazepam)	98.9	98.4	100	97.8	99.4	99.4	97.8	98.6	100
7.5 Quick-acting anti-seizure medication for seizures in pregnancy and post-partum (eg. im magnesium sulphate)	99.2	98.9	100	98.9	99.4	100	97.7	99.5	98.2
7.6 Cooling (eg. fans, antipyretics) in severe hyperthermia with a reduced level of consciousness.	93.5	93.0	94.7	92.3	94.1	93.6	93.3	93.2	94.6
8.1 Use of other indicated EECC treatments & actions if there is poor response to treatment, or if the patient deteriorates	98.8	98.9	98.6	98.9	98.8	98.2	100	98.5	100
8.2 Discontinuation of a treatment or action that is no longer indicated if a patient improves	94.7	93.6	97.4	91.4	96.5	94.8	94.4	96.1	89.5
8.3 Discontinuation of a treatment or action that has been deemed to no longer be in the patient’s best interest	94.7	94.7	94.7	92.4	96.0	94.3	95.6	96.2	89.5
8.4 Use of blankets and other means (including skin-to-skin care for babies) to keep the patient warm	99.2	98.9	100	100	98.8	99.4	98.9	99.5	98.2
8.5 Feeding (including breastfeeding for babies), naso-gastric feeding and dextrose for nutrition and to avoid hypoglycaemia	98.1	97.9	98.7	97.8	98.3	98.3	97.8	97.6	100
8.6 Treatment of pain and anxiety (eg. with needs-based psychological support, medication)	98.9	99.0	98.7	98.9	98.9	98.3	100	98.6	100
9.1 Infection, prevention and control (IPC) measures including hand hygiene and separation of patients with a suspected or confirmed contagious disease from those without	98.9	98.4	100	98.9	98.9	98.9	98.9	98.6	100
9.2 Seeking of assistance from additional or senior staff when a critically ill patient is identified	99.3	99.0	100	98.9	99.4	100	97.8	99.1	100
9.3 Care for all critically ill patients in locations that facilitate observation and care (eg. designated beds, a bay or a unit for critically ill patients)	99.6	99.5	100	100	99.4	99.4	100	99.5	100

9.4 Respectful and patient-centred care	99.3	100	97.5	97.9	100	98.9	100	99.1	100
9.5 Effective and respectful communication with the patient and family	99.3	100	97.5	97.9	100	98.9	100	99.1	100
9.6 Provision of EECC without considering the patient's ability to pay	97.0	96.8	97.4	95.7	97.7	96.6	97.8	96.7	98.3
9.7 Clear documentation of all treatments & actions	99.6	100	98.7	98.9	100	99.4	100	99.5	100
9.8 Recognition when EECC is not sufficient to manage the critical illness	98.9	98.4	100	97.9	99.4	98.9	98.9	99.5	96.5
9.9 Integration with care that is outside the scope of EECC (eg. the need for prompt investigations, definitive treatment of underlying conditions, end-of-life care, referral)	98.9	98.4	100	98.9	98.9	98.9	98.9	99.1	98.3
10.1 The EECC treatments & actions as specified for all critical illnesses	98.9	98.4	100	97.8	99.4	99.4	97.8	99.0	98.2
10.2 Personal Protective Equipment (PPE) that is appropriate for COVID-19 as part of Infection, Prevention and Control (IPC)	99.6	100	98.7	98.9	100	100	98.9	99.5	100
10.3 Monitoring oxygen saturation using pulse oximetry at least every 6 hours, unless otherwise prescribed	91.7	90.5	94.7	87.0	94.2	90.2	94.5	92.4	89.1
10.4 Intermittent prone positioning	90.9	88.5	97.1	94.1	89.3	94.6	84.1	89.4	96.4
10.5 Low molecular weight heparin or other anticoagulant	92.2	91.2	95.2	92.8	91.9	91.9	92.9	90.6	98.1
10.6 Corticosteroid	93.8	92.5	97.2	97.8	91.6	94.7	92.1	92.6	98.2
10.7 Antibiotics in patients with suspected bacterial superinfection	98.5	98.4	98.7	97.8	98.9	97.7	100	99.0	96.5

Supplementary Table 3 Subgroup analysis Round Two

Agreement (%) in subgroups for the treatments and actions that reached consensus in Round Two

Reached Consensus in Round 2	Delphi Panel	Intensive Care		Emergency Care		Low Income Country		Doctor	
**Treatments and Actions**		Intensive Care	Non-Intensive Care	Emergency Care	Non-Emergency Care	LICs	Non-LICs	Doctor	Non-doctor
1.16 Presence of respiratory distress (eg unable to complete sentences; accessory muscle use; chest recessions; grunting or head nodding)	93.4	95.2	88.7	96.3	91.8	94.4	91.0	92.5	97.6
1.17 Presence of severe dehydration (eg decreased skin turgor; dry mucous membranes; sunken fontanelle)	91.2	90.9	91.8	93.8	89.7	92.5	88.1	90.8	92.7
2.5 Less frequently for patients who have improved and are now stable (eg ...every 6hrs, every 12hrs, every 24hrs)	95.6	95.2	96.8	95.0	96.0	95.7	95.5	96.8	90.2
2.8 During and after transport/transfer of a patient who is critically ill or at risk of becoming critically ill	98.3	98.2	98.4	97.5	98.7	98.8	97.0	98.4	97.6
2.9 During and after surgery or anaesthesia	95.6	95.8	95.1	96.3	95.2	97.5	90.8	95.1	97.6
5.2 Oxygen therapy using nasal prongs	91.7	89.8	96.8	88.8	93.2	90.1	95.5	93.1	85.4
5.8 Bag-valve-mask ventilation for children	91.3	90.3	94.2	94.8	89.2	91.2	91.7	91.0	92.7
6.5 Oral rehydration solution or other appropriate oral fluids for dehydration without shock	94.3	94.6	93.6	94.9	93.9	93.2	97.0	94.7	92.5
6.6 Intramuscular adrenaline for anaphylaxis	90.6	91.3	88.7	92.3	89.7	88.6	95.4	92.0	83.3
6.9 Appropriate antibiotics for sepsis	97.4	97.6	96.7	97.5	97.3	96.9	98.5	97.3	97.6
6.10 Oxytocin when indicated	96.2	95.4	98.2	96.0	96.3	98.0	91.5	96.5	94.6
8.7 Mobilise the patient as early as possible	91.1	92.1	88.3	95.0	88.9	93.1	86.2	89.1	100
8.8 Insertion of an intravenous cannula when critical illness is identified	98.2	98.8	96.8	98.7	98.0	97.5	100	97.9	100
8.9 Insertion of an intraosseous cannula, if indicated, if an IV cannula is not possible	90.9	92.6	86.2	93.6	89.4	91.1	90.3	90.6	92.3
8.10 Regular turning of immobilised patients	94.3	94.0	95.2	93.8	94.6	94.4	94.0	93.1	100
8.11 Cervical spine stabilization in possible cervical spine injury	96.9	96.4	98.4	97.5	96.6	97.5	95.5	96.8	97.6
9.10 Clear communication and documentation about the planned EECC (eg continue oxygen therapy, give IV fluids	97.4	96.4	100	98.8	96.6	98.1	95.5	96.8	100

Schell CO, *et al. BMJ Global Health* 2022; 6:e006585. doi: 10.1136/bmjgh-2021-006585

Supplementary Table 4: Subgroup analysis Round Three

Agreement (%) in subgroups for the treatments and actions that reached consensus in Round 3

Reached Consensus in Round 3	Delphi Panel	Intensive Care		Emergency Care		Low Income Country		Doctor	
**Treatments and Actions**		Intensive Care	Non-Intensive Care	Emergency Care	Non-Emergency Care	LICs	Non-LICs	Doctor	Non-doctor
1.8 Temperature	93.3	92.8	94.6	94.0	92.9	93.5	92.9	93.1	94.3
1.9 Capillary refill time	94.8	94.9	94.4	97.0	93.7	95.6	92.9	93.6	100
1.12 Confused, agitated or disoriented mental state	94.3	94.2	94.6	97.0	92.9	94.9	92.9	93.1	100
1.15 Presence of a generalized seizure	91.1	89.1	96.3	95.4	88.9	94.9	81.8	89.7	97.1
1.18 Inability to breastfeed or feed in a young child	92.9	92.4	94.1	90.6	94.1	93.8	90.7	92.6	94.1
2.3 For hospital in-patients, at least every 24 hours, unless otherwise prescribed (Note: can be done more frequently)	96.4	95.7	98.2	95.5	96.9	95.7	98.2	96.2	97.1
2.7 When a patient, family member or guardian is concerned that the patient may be critically ill	94.8	94.9	94.6	94.0	95.2	95.7	92.7	95.6	91.4
5.9 Bag-valve-mask ventilation for adults	96.8	96.4	98.1	98.5	95.9	96.3	98.2	96.1	100
9.11 Prevent delirium (eg. sleep hygiene, provision of the patient's glasses or hearing aid)	91.6	92.1	90.2	90.8	92.0	91.2	92.6	91.0	94.3
